# A systematic review describing incidence rate and prevalence of dysvascular partial foot amputation; how both have changed over time and compare to transtibial amputation

**DOI:** 10.1186/s13643-017-0626-0

**Published:** 2017-11-21

**Authors:** Michael P Dillon, Matthew Quigley, Stefania Fatone

**Affiliations:** 1 Discipline of Prosthetics and Orthotics, College of Science, Health and Engineering, La Trobe University, Melbourne, 3086 Australia; 2 Northwestern University Prosthetics-Orthotics Center, Feinberg School of Medicine, Northwestern University, 680 N Lake Shore Drive, Suite 1100, Chicago, IL 60611 USA

**Keywords:** Amputation, Partial foot, Transtibial, Incidence, Prevalence, Epidemiology

## Abstract

**Background:**

Partial foot amputation (PFA) is a common consequence of advanced peripheral vascular disease. Given the different ways incidence rate and prevalence data have been measured and reported, it is difficult to synthesize data and reconcile variation between studies. As such, there is uncertainty in whether the incidence rates and prevalence of PFA have increased over time compared to the decline in transtibial amputation (TTA). The aims of this systematic review were to describe the incidence rate and prevalence of dysvascular PFA over time, and how these compare to TTA.

**Method:**

Databases (i.e., MEDLINE, EMBASE, psychINFO, AMED, CINAHL, ProQuest Nursing and Allied Health) were searched using MeSH terms and keywords related to amputation level and incidence rate or prevalence. Original research published in English from 1 January 2000 to 31 December 2015 were independently appraised, and data extracted, by two reviewers. The McMaster Critical Review Forms were used to assess methodological quality and bias. Results were reported as narrative summaries given heterogeneity of the literature and included the weighted mean annual incidence rate and 95% confidence interval.

**Results:**

Twenty two cohort studies met the inclusion criteria. Twenty one reported incidence rate data for some level of PFA; four also included a TTA cohort. One study reported prevalence data for a cohort with toe(s) amputation. Samples were typically older, male and included people with diabetes among other comorbidities. Incidence rates were reported using a myriad of denominators and strata such as diabetes type or initial/recurrent amputation.

**Conclusion:**

When appropriately grouped by denominator and strata, incidence rates were more homogenous than might be expected. Variation between studies did not necessarily reduce confidence in the conclusion; for example, incidence rate of PFA were many times larger in cohorts with diabetes (94.24 per 100,000 people with diabetes; 95% CI 55.50 to 133.00) compared to those without (3.80 per 100,000 people without diabetes; 95% CI 1.43 to 6.16). It is unclear whether the incidence rates of PFA have changed over time or how they have changed relative to TTA. Further research requires datasets that include a large number of amputations each year and lengthy time periods to determine whether small annual changes in incidence rates have a cumulative and statistically significant effect over time.

**Systematic review registration:**

PROSPERO CRD42015029186.

**Electronic supplementary material:**

The online version of this article (10.1186/s13643-017-0626-0) contains supplementary material, which is available to authorized users.

## Background

Lower limb amputation is a common consequence of advanced peripheral vascular disease, often secondary to the long-term consequences of diabetes [1]. Unfortunately, little is known about the number people living with limb loss given the paucity of prevalence data and uncertainty inherent in estimating prevalence based on historical trends in amputation incidence and mortality [2]. By comparison, there is a comparatively large body of literature that suggests the incidence rate of lower limb amputation has remained fairly constant over the last 15 years [3, 4]. A more detailed look at these data suggests there may have been a shift in the types of lower limb amputations performed [3–7]. The incidence rate of transtibial amputation (TTA) seems to have declined [3, 8–11], while there is some evidence that the incidence rate of partial foot amputation (PFA) has increased proportionately [3, 7].

There is considerable uncertainty in these observations given the different ways these data have been measured, standardized, and reported. For example, studies have expressed the number of amputations as a function of the total population (e.g., per 100,000 population), an at-risk population (e.g., per 10,000 people with diabetes), or as a true rate that accounts for the time people are at-risk (e.g., per 1000 person-years) [12]. Such variation in the incidence rate data are further complicated depending on which amputation procedures are counted. For example, some studies exclude people with toe amputations [13] and as such, likely underestimate the true incidence rate of PFA given that about 60% of PFA affect one or more toes [3, 14].

While the effect of these sorts of variations in method design have been discussed in the literature [12, 15, 16], the extent to which they actually explain variation in the incidence rates has not been scrutinized in the context of PFA and TTA.

A systematic review of recent epidemiological research would provide a means to make sense of the various ways these incidence rate data have been reported and where possible, synthesize these data to describe the incidence rate and understand how this has changed over time in people with PFA compared to TTA. Critical appraisal of the method design would help explain variation in the incidence rates between studies and help reconcile the seemingly disparate data reported in the literature.

A more informed understanding of the incidence rate and prevalence data are important to establish how incidence rates and prevalence may have changed over time so that we can plan for the specialist health care needs of those facing the prospect of, and living with, PFA.

Therefore, the aims of this systematic review were to:

1. describe the incidence rate and prevalence of dysvascular PFA,
2. describe whether the incidence rate and prevalence of dysvascular PFA has changed over time,
3. describe causes of variation in the incidence rate and prevalence reported,
4. compare the incidence rate and prevalence of dysvascular PFA and TTA.

## Methods

Prior to conducting this review, a detailed systematic review protocol was registered in PROSPERO (CRD42015029186) and published [17]; hence, a summary of the methods related to the epidemiological review have been reported here. We highlight that the protocol also included aims related to the outcomes of amputation which have been published in another systematic review [1].

### Search strategy

A search of the literature was systematically conducted using MEDLINE, EMBASE, psychINFO, AMED, CINAHL, ProQuest Nursing and Allied Health. Search terms related to the population and outcomes of interest were used in conjunction with wildcards and Boolean operators as part of a title, abstract, and keyword search [17]. Each search strategy was developed, tested and refined by comparing the precision and comprehensiveness of the articles retrieved to a bank of known articles on the topic [17].

All searches were limited to articles written in English given that such language restriction does not alter the outcome of systematic reviews and meta-analyses [18, 19]. The search was restricted to publications since 1 January 2000 given that changes in treatment practices (e.g., common place use of revascularization prior to, or in conjunction with, amputation) have affected outcomes over time [8, 10, 11].

Consistent with the Preferred Reporting Items for Systematic Reviews and Meta-Analyses (PRISMA) guidelines, [20] an illustrative search for one database is shown in Table 1.

**Table 1:** Example search for the CINAHL database to identify incidence and prevalence literature for people with dysvascular partial foot and transtibial amputation

Search	Field code	Search term(s)
1.	MH	“Amputation”
2.	MH	“Amputees”
3.	TI,AB,SU	(amput* AND (major OR lowerlimb* OR “lower limb”* OR “lower extremit*” OR “limb loss” OR LEA OR LLA))
4.	TI,AB,SU	(amput* AND (transtibial OR “trans tibial” OR belowknee OR “below knee” OR (below W2 knee) OR TTA OR BKA))
5.	TI,AB,SU	(amput* AND (minor OR “partial foot” OR Chopart* OR Lisfranc* OR tarsometatarsal OR transmetatarsal OR midtarsal OR “mid tarsal” OR midfoot OR “mid foot” OR ray OR phalangeal OR metatarsophalangeal OR toe* OR transtarsal OR “trans tarsal” OR TMT OR TMA OR MTP OR PFA))
6.		1 OR 2 OR 3 OR 4 OR 5
7.	TI,AB,SU	“incidence” OR “rate” OR “incidence rate” OR “prevalence” OR “trend”
8		6 AND 7
9.		Limit 8 to English language
10.		Limit 9 to publication date: 01 January 2000 to 31 Decemeber 2015
11.		Limit 10 to peer reviewed, academic journals

Field codes: *MH* exact major and minor subject headings (MeSH, National Library of Medicine Medical Subject Headings), *TI* title, *AB* abstract, *SU* subject

Reference lists of included articles were hand searched to ensure that relevant publications were not missed. A forward-citation search using Google Scholar was conducted to identify early on-line articles published since the 1 January 2014 that had not yet been indexed in traditional databases [21–23].

### Data management

Search results from each database were exported into a shared EndNote X7.2.1 library (Thomson Reuters Inc., Philadelphia, PA, USA.) and duplicate records deleted [17]. Full-text articles were retrieved and linked to the corresponding EndNote record. Bibliographic information were exported into an Excel 2013 (Microsoft Corporation Inc., North Ryde, Sydney, Australia) spreadsheet to track details about exclusion and full-text retrievals. The same spreadsheet was expanded for data extraction and critical appraisal [17].

### Selection process

Inclusion criteria were as follows:

1. Peer reviewed studies of original research, irrespective of their design;
2. English language;
3. Published between 1 January 2000–31 December 2015;
4. Discrete cohort(s) with either: dysvascular PFA (irrespective of the level of PFA) or dysvascular PFA and TTA; and
5. Reported data on the incidence rate or prevalence.

The International Standards Organization (ISO) definitions [24] of TTA and PFA were used. As such, all levels of PFA (including amputation of one or more toes) were included but ankle disarticulation (i.e., Syme’s amputation) was excluded. Articles were included regardless of the way the numerator or the denominator were operationally defined [17].

Search results were screened by one investigator based on review of the title, abstract, or full-text as necessary. After screening, all full-text articles were retrieved and reviewed independently by two investigators to confirm inclusion. The opinion of a third investigator was sought in cases of disagreement, and discussion occurred until consensus was achieved.

### Quality appraisal/risk of bias in individual studies

Methodological quality and sources of bias were assessed using the McMaster Critical Review Forms [25, 26] given this study formed part of a larger review into the outcomes of PFA and TTA [1] that included studies of various designs [27]. The McMaster Critical Review Forms include structured guidelines to reduce the likelihood of errors with their use [28]. The quality appraisal was collated using Excel with detailed comments included to support the checklist items [17].

### Data extraction

Socio-demographic, methodological, results, and quality appraisal details were recorded in an Excel spreadsheet (Additional file 1) for each included article using the Cochrane Consumers and Communication Review Group’s data extraction template [29]. The data extraction spreadsheet was piloted and refined prior to use [17].

Two reviewers independently appraised included articles. Data were extracted from each article by a primary reviewer and checked by a second reviewer for accuracy and clarity. A third reviewer was called upon to appraise the article and contribute to the consensus decision as necessary. Authors of the original research were contacted for additional information or to clarify method details. Reminder emails were sent if a response was not received.

In cases where incidence rate or prevalence data were reported in figures only, authors of the original research were contacted to obtain these data. Where we were unable to obtain these data from authors, we utilized software (DigitizeIt v 2.2.2, www.digitizeit.de) to digitize the figures and extract the *x*, *y* coordinates. This approach has been shown to be valid and reliable in several studies, and we adopted recommendations for minimizing errors, such as zooming in to identify the center of data points [30–32].

For articles where data for the same participants were reported, subject numbers, demographics, and outcomes were compared across studies for discrepancies. Any uncertainty about the similarity in study participants and results, were clarified by contacting the authors of the original research. Where the same subjects were included in multiple studies, data were treated as a single source but all studies were cited.

### Data summary and reporting

Extracted data were explored to identify variation in the way the prevalence or incidence rate data were reported and, where possible, efforts were made to reduce apparent variation. For example, studies that expressed the incidence rate per 10,000 or 100,000 people with diabetes were scaled to a common denominator (e.g., per 100,000 people with diabetes) to reduce variance and facilitate synthesis. Results were presented in separate sections for each denominator (e.g., per 100,000 people with diabetes) and included various subgroup analyses (e.g., stratified by diabetes type) with a view to synthesizing like data while preserving information inherent in the different strata reported. As part of the narrative review, issues with internal and external validity that most influenced the incidence rates or prevalence were discussed with a specific focus on limitations that lead to imprecision and heterogeneity [33].

Where possible, descriptive statistics were used to summarize data. A mean annual incidence rate was calculated for each of the included studies; using the age- and sex-standardized incidence rates in preference to the crude incidence rates where possible. Notable variation between the crude or age- and sex-standardized incidence rates were highlighted through the narrative given the results were influenced by the population structure. To synthesize data across studies, the mean annual incidence rates were weighted by the average number of amputations each year to produce a point estimate and 95% confidence interval using StatsDirect3 (StatsDirect Limited, Cheshire, UK, www.statsdirect.com).

## Results

### Study selection

**Fig. 1:**
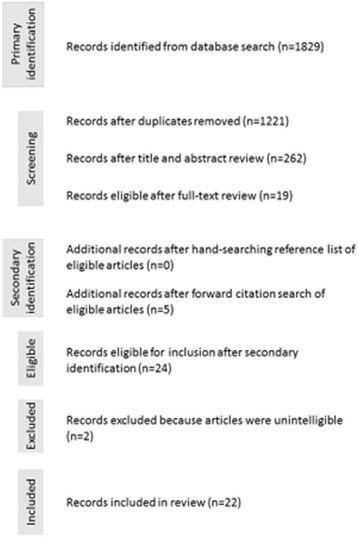
PRISMA eligibility flowchart

The search initially yielded 1829 articles (Fig. 1). Following removal of duplicates, 1221 were vetted against the inclusion criteria based on title and abstract, leaving 262 articles for full-text review. Of these, 19 articles met the inclusion criteria. Hand-searching the reference lists of these 19 articles did not yield any additional articles. Forward citation searching identified an additional five articles that met the inclusion criteria. However, of the 24 articles that met the inclusion criteria, 2 were unintelligible and therefore unable to be included [34, 35], leaving a total of 22 included articles.

### Study characteristics

Studies included in the review arose from many different countries, with a number from Australia [36–38], Spain [39–42], England [7, 43–47], and the USA [2, 13, 48, 49]. Isolated studies included populations from Italy [9], Ireland [50], Scotland [51], Sweden [52], and Taiwan [53].

All studies were cohort designs, and most used retrospective data sourced from hospital [44–46, 52], Veterans Health Administration, state [13, 37–39, 43, 48, 49] or national [2, 7, 9, 40–42, 47, 50, 51, 53] databases. However, variation in nomenclature across juristictions made it difficult to be certain about this categorization without intimate knowledge of the individual heathcare settings. Only a few studies collected data prospectively [36, 45] or used retrospective data to supplement, and thereby extend the time period [44].

The size of the cohorts with PFA varied markedly, partly because the study time frames varied (median 7 years, range 1–18 years). Several studies included PFA cohorts with less than 100 people typical of individual healthcare services that averaged fewer than 10 amputations per annum [36, 45, 46, 52]. By contrast, the largest studies included PFA cohorts with tens of thousands of people [9, 41, 42, 47] typical of large national datasets. When averaged over the time frames, these large studies included thousands [9, 41, 42, 47] of PFAs per annum. Three studies did not report the number of people in the PFA cohort, and we were unable to obtain these data from the authors [7, 43, 44].

By virtue of the inclusion criteria, all studies reported data for people with dysvascular PFA, while a few studies included separate cohorts with PFA and TTA [13, 36, 48, 49]. Given the population of interest, it was not suprising that studies included samples that were typically older, male, and included people with diabetes among other comorbidities (Table 2). Unfortunately, a number of studies did not report these basic demographic data [44, 45, 50, 51]. In other cases, these demographic data were not reported by cohorts with PFA or TTA to match the incidence rate data reported [7, 9, 38–40, 42, 43, 47, 48, 53] but rather by groups suited to the particular study aims (e.g., lower limb amputee cohort with and without diabetes). As such, it was often difficult to determine the representativeness of the PFA and TTA cohorts.

**Table 2:** Demographic information

Author		Partial foot amputation				Transtibial amputation				A	B	C	D	E	F	G	H	I	J	K	L	M	O
	Years	(n)	DM	Male	Age	(*n*)	DM	Male	Age														
			(%)	(%)	M(SD)		(%)	(%)	M(SD)														
Almaraz et al. [39]	9	7007	73^a^	66^a^	68.6(11.5)^a^										X								
Alvarsson et al. [52]	6	46	76	55^a^	81^a^	109	62	55^	81^						X				X		X		X
Buckley et al. [50]	5	NR	54^a^	NR	NR																		
Canavan et al. [43]	5^b^	NR	49^a^	66^a^	73% < 60					X		X			X							X	X
Davis et al. [36]	9	43	100	59^c^	66.8(9.3)	13	100	59^c^	66.8(9.3)					X	X	X	X	X	X	X	X		X
Dillingham et al. [48]	12	8684^d^	43^a^	54^a^	67.7(14.7)^a^	6883	43^a^	54^a^	67.7(14.7)^a^	X					X	X							X
Feinglass et al. [13]	18	5733^e^	80	65	64.9(12.9)	14,801	70	56	68.8(12.8)	X	X				X								X
Kennon et al. [51]	5	1023	NR	NR	NR																		
Krishnan et al. [44]	11	NR	NR	NR	70.7^a^																		
Kurowski et al. [38]	11	3796	64^a^	66^a^	65^a^					X			X		X		X	X		X	X		X
Lai et al. [53]	11	776	100	NR	NR																		
Lazzarini et al. [37]	6	3009	100	71	64(median)																		
Lombardo et al. [9]	8	51,253	68^a^	NR	71.1(11.1)^f^																		
Lopez-de-Andres et al. [41]	7	46,536	80	74	66.9(14.6)										X								
Lopez-de-Andres et al. [42]	11	73,302	75	75	66										X	X							
Rayman et al. [45]	3	34	100	NR	NR																		
Rubio et al. [40]	11	248	73	69^g^	71^g^																		
Sandnes et al. [49]	14	4434	66	61	66(15.5)										X	X				X			
Valabhji et al. [46]	5	34	NR	76	68(11)										X				X	X	X		
Vamos et al. [47]	10	S^h^	S^h^	S^h^	S^h^						X				X								
Vamos et al. [7]	5	S^h^	S^h^	S^h^	S^h^						X				X								
Ziegler-Graham et al. [2]	1	NR	NR	NR	NR																		

*Years* years included in timeseries; *DM* diabetes mellitus*; M(SD)* mean (standard deviation); *PFA* partial foot amputation*; TTA* transtibial amputation; *NR* not reported; *A* race/ethnicity; *B* sociodemographic: include education level, relationship status, employment status, residential status, geographic region, economic status; *C* tobacco use; *D* other lifestyle behaviors include alcohol use and malnutrition; *E* BMI: body mass index, *F* diabetes, *G* comorbidities include the Charlson comorbidity index, frequency of comorbidities and the Anesthesiology Association of America Physical Status scale; *H* blood pressure; *I* other blood flow/pressure issues: include missing pulses, ankle brachial index, Doppler for ankle blood pressure; *J* coronary artery disease or cerebrovascular accident; *K* other cardiovascular problems include cardiovascular disease, myocardial infarct, heart attack, peripheral arterial disease, peripheral vascular disease, and chronic obstructive pulmonary disease; *L* renal issues include nephropathy, renal dialysis, chronic renal failure, renal insufficiency, and end-stage renal disease; *M* reamputation; *O* other includes ulcer, diabetes with end organ damage, and zip code

^a^Based on lower limb sample as a whole

^b^Duration of study: 5 years; data reported for 3 years

^c^For those with diabetes-related amputation

^d^Toe level only; ‘foot’ amputations excluded due to inclusion of ankle disarticulation

^e^‘Through foot’—excludes toe only amputations

^f^For people with diabetes in 2010

^g^Weighted average

^h^Stratified by proportions with and without diabetes; this information is presented in full in the appendix

Epidemiological data were presented in a myriad of ways. For example, incidence rates were typically reported as either crude [36, 39, 44–46, 51, 53] or standardized by either age [43, 50] or, more commonly, age and sex [13, 38, 40–42, 47–49]. Two studies reported both the crude and standardized incidence rates [9, 37]. Incidence rates were often reported by diabetes type [38, 40–42, 47] and less commonly by race [48] or initial/recurrent amputation [38]. Incidence rates were expressed per 100,000 general population [13, 39–42, 44–48, 51], 100,000 person-years general population [37, 38, 49], 1000 patient-years [36], 100,000 people without diabetes [9, 39, 50], 1000 person-years with diabetes [37] or 1000, 10,000 or 100,000 people with diabetes [9, 39, 43, 45, 46, 50–53].

Given an understanding of the myriad ways these data were reported, results have been presented in subsections by denominator (e.g., per 100,000 population). Within each of these subsections, data have been presented for each strata (e.g., stratified by diabetes type) and over time.

### Incidence rate of PFA per 100,000 general population

The incidence rate of PFA per 100,000 general population were reported in 11 studies [13, 39–42, 44–48, 51]. The two studies by Lopez-de-Andres et al. [41, 42] included the same samples and, as such, have been treated as a single data source using results from the latter of these publications [42] given that it included all data from the preceding publication [41].

Five studies reported the incidence rate of PFA per 100,000 general population without stratification [13, 44–46, 51]. While the mean annual incidence rate reported by Krishnan et al. [44] was similar to the other studies (Table 3), the total number of amputations over the time series were not reported and, as such, the study could not be included in the weighted mean annual incidence rate. Based on the remaining four studies [13, 45, 46, 51], the weighted mean annual incidence rate was 4.0 per 100,000 general population (95% CI, 3.82 to 4.17). The homogeneity of the incidence rates reflects the similarity of the method designs [13, 44–46, 51]. As an illustrative example, repeat amputations within the first few months [44–46, 51] or within the same admission [13] were counted as a single procedure at the highest amputation level. The true incidence rate per 100,000 general population is likely to be much higher given the large proportion of people that progress to another amputation within the first few weeks or months after PFA [1, 54].

**Table 3:** Mean annual incidence rate of partial foot amputation per 100,000 general population

Study	Time period	Strata	Standardization			Annual Incidence rate		Amputations per annum
			Crude	Age	Sex	Mean	SD	Mean
No Stratification								
Almaraz et al. [39]	1998–2006		Yes	No	No	17.42	0.94	779
Feinglass et al. [13]	1987–2004		No	Yes	Yes	4.02	0.85	319
Kennon et al. [51]	2003–2008		Yes	No	No	4.00	0.31	205
Krishnan et al. [44]	1995–2005		Yes	No	No	3.49	1.15	NR
Valabhji et al. [46]	2004–2009		Yes	No	No	3.90	4.90	7
Rayman et al. [45]	1997–1999		Yes	No	No	3.37	0.99	11
Diabetes presence								
Rubio et al. [40]	2001–2011	DM	No	Yes	Yes	5.48	1.89	17
		No DM	No	Yes	Yes	1.46	0.48	5
Diabetes type								
Vamos et al. [47]	1996–2005	Type 1 DM	No	Yes	Yes	1.65	0.21	575
		Type 2 DM	No	Yes	Yes	3.10	0.59	1190
Lopez de Andres et al. [42]	2001–2012	Type 1 DM	No	Yes	Yes	0.60	0.15	236
		Type 2 DM	No	Yes	Yes	10.82	0.61	4324
Lopez de Andres et al. [41]	2001–2008	Type 1 DM	No	Yes	Yes	0.64	0.15	250
		Type 2 DM	No	Yes	Yes	10.41	0.38	4023
Race								
Dillingham et al. [48]	1986–1997	Black	No	Yes	Yes	19.00	1.50	246
		Non-black	No	Yes	Yes	12.78	1.27	477

*SD* standard deviation, *DM* diabetes mellitus, *NR* not reported

One study reported the incidence rate of PFA per 100,000 general population without stratification in those over 30 years of age [39]. This variation in the method design resulted in a 4-fold increase in the incidence rate compared to other studies where the denominator included all people in the population [13, 44–46, 51], not just those over 30 years of age (Table 3). While this publication may better describe the true incidence rate in the population at risk, it could not be synthesized with other studies that reported the incidence rate per 100,000 general population without stratification [13, 44–46, 51] given the different method designs.

One study reported the incidence rate of PFA per 100,000 general population stratified by the presence (or absence) of diabetes [40]. Using a national surgical procedures register, people with diabetes were identified as those having an ICD-9-CM code for diabetes, irrespective of the type. Those without the code were included in the cohort without diabetes and, as such, the reliability of the coding was key. Given the study focused on a specific health district in Mardrid, the average number of amputations per year was small (< 20) and the annual incidence rates fluctuated as a result. When averaged over time, the mean annual incidence rate was 30% lower in the cohort without diabetes compared to the cohort with diabetes. While internally valid, it was not clear why the incidence rates were subsequently standardized to the European population and, with only details about age and sex reported, and the small sample, it was difficult to be confident in the external validity of the study.

Three studies reported an incidence rate of PFA per 100,000 general population stratified by diabetes type [41, 42, 47]. While these studies all report higher incidence rates in people with type 2 diabetes, there was considerable variation between studies. In comparison to those with type 1 diabetes, the mean annual incidence rate for those with type 2 diabetes was 18-fold larger in one study [41, 42], but only 2-fold larger in another [47]. Such large variations in the incidence rates were difficult to reconcile given these studies used similar national health data sets and inclusion criteria to identify amputation discharges, comparable ICD codes to identify those with different types of diabetes, and national population statistics as the denominator. We have some concern about the quality of the data in the study by Vamos et al. [47] given that the mean annual incidence rate was two times larger in the non-diabetic group compared to the group with type 2 diabetes [47], which was inconsistent with other studies [9, 39–41, 50].

One study reported an incidence rate of toe amputation per 100,000 general population stratified by race; denoted in the article as ‘black’ or ‘non-black’ [48]. The black cohort included African American people while the non-black cohort included ‘primarily white people’ given that only 1.5% of the cohort were from diverse racial and ethnic groups (not defined) [48]. In comparison to the black cohort, the mean annual incidence rate was one-third lower in the non-black cohort [48] (Table 3); acknowledging that the cohorts were similar in terms of mean age and proportions with diabetes and peripheral vascular disease. These results may be difficult to generalize to other populations in the USA, or indeed other countries, with more racially diverse populations. While Dillingham et al. [48] also reported incidence rate data for a ‘foot’ cohort (i.e., partial foot), these data were not appropriate for inclusion given the foot cohort included people with ankle disarticulation.

**Fig. 2:**
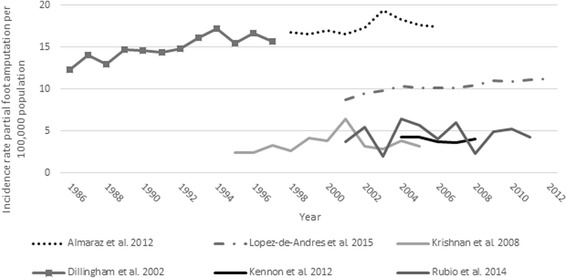
Incidence rate of partial foot amputation per 100,000 general population over time

Six studies were designed to test whether the incidence rate of PFA per 100,000 general population changed over time [39–42, 48, 51]. Four studies used appropriate inferential analysis techniques including: Poisson [39], joinpoint [40, 42], or linear [48] regression models. Results were often stratified by the presence [39] or type of diabetes [42] or race [48]. Given these variations in method design, changes in the incidence rate over time differed depending on the strata reported. When the incidence rate of PFA was stratified by the presence/absence of diabetes, it increased only in those with diabetes [39]. When considered with respect to the type of diabetes, there was a significant linear increase in the incidence rate of PFA in a cohort with type 2 diabetes (2001–2012), but a significant descrease in those with type 1 diabetes between 2001 and 2008 before plateauing [42]. In terms of race, incidence rates increased significantly over time in both ‘black’ (i.e., African Americans) and ‘non-black’ (i.e., prediminently white) cohorts [48] (Fig. 2).

### Incidence rate of PFA per 100,000 person-years (general population)

The incidence rates of PFA per 100,000 person-years (general population) were reported in two studies [37, 49]. The weighted mean annual incidence rate was 10.42 per 100,000 person-years general population (95%CI − 14.76 to 35.61). Given how similar the method designs were between these studies (e.g., both used state-wide surgical data sets and national census data requiring similar assumptions and standardization), variation in the incidence rates likely reflects differences in the sample characteristics. For example, Lazzarini et al. [37] only included people with ICD codes for amputation and diabetes, resulting in a sample with a higher proportion of males and people with diabetes compared to Sandnes et al [49] (Table 4).

**Table 4:** Mean annual incidence rate of partial foot amputation per 100,000 person-years general population

Study	Time period	Strata	Standardization			Annual incidence rate		Amputations per annum
			Crude	Age	Sex	Mean	SD	Mean
No stratification								
Lazzarini et al. [37]	2005–2010		Yes	No	No	12.06	0.78	502
			No	Yes	Yes	12.06	0.86	502
Sandnes et al. [49]	1986–2000		No	Yes	Yes	7.93	1.06	317

SD: standard deviation

**Fig. 3:**
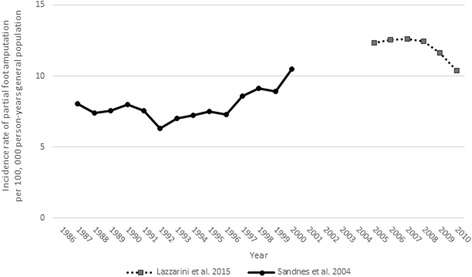
Incidence rate of partial foot amputation per 100,000 person-years general population

Both of these studies [37, 49] tested for changes in the incidence rates over time using common inferential analysis techniques including the chi-square test for trend [37] or Poisson regression [37, 49]. While these statistical approaches may be common for the analysis of epidemiological data, results from these studies highlight that the choice of inferential analysis can result in different conclusions. Recognizing this challenge, Lazzarini et al. [37] used two different inferential techniques to test whether the 15.7% reduction in the age- and sex-standardized incidence rates of PFA from 2005 to 2010 (Fig. 3) was statistically significant. While the reduction in the incidence rate over time was statistically significant, the chi-squared test for trend assumes changes over time are linear and as such, the statistical approach was not robust for use in this study given the curvilinear decline in the incidence rate over time (Fig. 3). By contrast, use of Poisson regression with the same data showed that the incidence rates were not significantly different year-to-year and that only differences between the first and last year in the time series were statistically significant [37] (Fig. 3). While this example suggests that use of the chi-squared test led to a spurious finding, and that Poisson regression might have been a more appropriate statistical approach in this instance, it is important to recognize that the choice of statistical test is more complex. For example, while Sandnes et al. [49] also used the Poisson regression and found no statistically significant change over time, we hypothesize that significant changes in the incidence rates during the time series may have been missed given the descriptive data presented (Fig. 3). Notwithstanding the year-to-year variability in the data, we hypothesize that the use of a joinpoint regression model may have better characterized the large and linear increase in the incidence rates from 1992 to 2000 (Fig. 3).

### Incidence rate of PFA per 100,000 person-years (population at risk)

Three studies reported an incidence rate of PFA using a variety of denominators including per 100,000 person-years (population at risk) [38], per 1000 patient-years [36], and per 1000 person-years with diabetes [55]. Given that the populations at-risk in these three studies were all people with diabetes, it was possible to express the incidence rates using a common denominator (i.e., per 100,000 person-years population at risk).

The use of a common denominator served to reduce variance in the numerators that made it easier to see the similarirty in the incidence rates across studies. For example, two studies reported notably similar incidence rates of PFA per 100,000 person-years (population at risk) (Table 5), acknowledging that one stratified by level of PFA [36] and the other did not [37]. The incidence rate of toe amputation was 10-fold larger than all other levels of PFA combined, highlighting the impact that counting (or excluding) toe amputations can have on the incidence rates of PFA reported [36].

**Table 5:** Mean annual incidence rate of partial foot amputation per 100,000 person-years (population at risk)

Study	Time period	Strata	Standardization			Annual incidence rate		Amputations per annum
			Crude	Age	Sex	Mean	SD	Mean
No Stratification								
Lazzarini et al. [37]	2005–2010		Yes	No	No	361	61	502
Davis et al. [36]	1996–2000	Toe	Yes	No	No	340	NR	10
		Foot	Yes	No	No	30	NR	0.5
Kurowski et al. [38]	2000–2010	T1 DM initial	No	Yes	Yes	257	76	12
		T1 DM repeat	No	Yes	Yes	5908	2704	11
		T2 DM initial	No	Yes	Yes	238	21	124
		T2 DM repeat	No	Yes	Yes	23,011	5888	86

*SD* standard deviation, *T1 DM* type 1 diabetes mellitus, *T2 DM* type 2 diabetes mellitus, *NR* only mean annual incidence rate data reported (without standard deviation). No standard deviation able to be calculated given annual incidence rate data not reported over time

The third study that reported the incidence rate of PFA per 100,000 person-years (population at risk) stratified by diabetes type and initial/recurrent amputation [38]. The mean annual incidence rates of initial PFA were similar in cohorts with type 1 (257 per 100,000 person-years population at risk) and type 2 diabetes (237 per 100,000 person-years population at risk), which suggests that the higher incidence rates typically associated with type 2 diabetes [42, 47] may be confounded by inclusion of people with first and recurrent amputation in the same cohort. The confounding influence of recurrent amputation on diabetes type is self-evident when you consider that, in comparion to initial PFA, the mean annual incidence rate of recurrent PFA was 23 times higher in people with type 1 diabetes and 100 times higher in people with type 2 diabetes [38] (Table 5). The very high incidence rates of recurrent PFA is a reflection of the large proportion of people that progress to another amputation with the first months or years after PFA (numerator), and that the years at-risk (i.e., at-risk of dying) is small given the high mortality rates following PFA (denominator) [1].

**Fig. 4:**
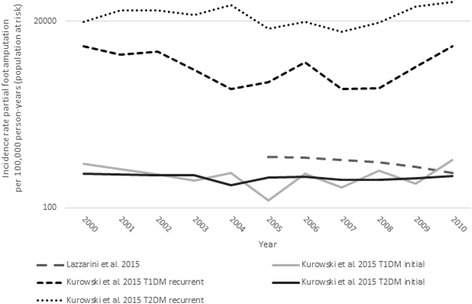
Incidence rate of partial foot amputation per 100,000 person-years (population at risk)

Two studies reported changes in the incidence rate of PFA per 100,000 person-years (population at risk) over time (Fig. 4) using either a chi-squared test for trend [37] or a Poisson log-linear regression model [38]. Lazzarini et al. [37] reported a 37.5% reduction in the incidence rate from 2005 to 2010, which was statistically significant (Fig. 4). In comparison, Kurowski et al. [38] reported no change in the incidence of initial PFA in groups with type 1 or type 2 diabetes, and a statistically significant increase in recurrent PFA in people with type 2 diabetes over the time series [38] (Fig. 4). The contrasting results between these studies likely reflects the different way diabetes prevelance were estimated. Kurowski et al. [38] estimated diabetes prevelance for each year of the time series based on a 15 year look-back period whereby individual hospital records were searched for diabetes related ICD codes using a comprehensive state-wide linked data system. By contrast, Lazzarini et al. [37] estimated diabetes prevalence using data from the Australian National Diabetes Services Scheme; a federal scheme designed to support people with diabetes to manage their care and access free or subsidized products such as insulin pen needles. Over the 6-year time series Lazzarini et al. [37] estimated that the diabetic population increased by 56%, which is about three times larger than that observed in another Australian state with an annual prevalence surveilance system in place [56]. As decribed by Lazzarini et al. [37], the reduction in the incidence rate of PFA per 100,000 person-years (population at risk) is likely to be exaggerated given the increased rate of diagnosis of diabetes and the rapid rate of registration with the National Diabetes Services Scheme over the time period. Based on other studies that have also reported incidence rates using both a diabetic population denominator as well as a general population denominator [10, 11], we contend that any change in the incidence rate over time is likely to be more akin to that previously reported in this review per 100,000 general population and as such, annual reductions in the incidence rate of PFA are likely to be small and only statisticaly significant over many years.

### Incidence rate of PFA per 100,000 people with and without diabetes

Four studies reported the incidence rate of PFA per 100,000 people with and without diabetes [7, 9, 39, 50].

One study reported the incidence rate of PFA per 100,000 people over 30 years of age with and without diabetes (Table 6) [39]. As previously discussed, this approach may better reflect the population at risk, but makes it inappropriate to synthesize these data with studies that include all people, not just those over 30 years of age.

**Table 6:** Mean annual incidence rate of partial foot amputation per 100,000 people with and without diabetes

Study	Time period	Strata	Standardization			Annual incidence rate		Amputations per annum
			Crude	Age	Sex	Mean	SD	Mean
Without diabetes								
Almaraz et al. [39]	1998–2006		Yes	No	No	3.36	0.39	141
Buckley et al. [50]	2005–2009		No	Yes	No	4.56	0.33	121
Lombardo et al. [9]	2003–2010		Yes^a^	Yes	Yes	3.75	0.24	2051
Vamos et al. [7]	2004–2008		No	Yes	Yes	5.16	0.40	NR
With diabetes								
Almaraz et al. [39]	1998–2006		Yes	No	No	247.63	19.96	638
Buckley et al. [50]	2005–2009		No	Yes	No	108.00	8.49	214
Lombardo et al. [9]	2003–2010		Yes*	Yes	Yes	93.56	4.94	4355
Vamos et al. [7]	2004–2008		No	Yes	Yes	151.60	3.44	NR

^a^While both crude and age-sex-standardized data reported in the study, data reported here reflects age-sex-standardized data

The remaining three studies reported the incidence rate of PFA per 100,000 people with and without diabetes [7, 9, 50]. While the mean annual incidence rates of PFA were similar across these three studies [7, 9, 50] (Table 6), Vamos et al. [7] did not report the total number of amputations over the time series and as such, we were unable to include these data in our calculation of a point estimate and 95% confidence interval. Based on the remaining two studies, the weighted mean annual incidence rate of PFA was estimated to be 25 times higher in those with diabetes (94.24 per 100,000 people with diabetes; 95% CI 55.50 to 133.00) compared to those without (3.80 per 100,000 people without diabetes; 95% CI 1.43 to 6.16), which was similar to the increased risk of PFA in people with diabetes calculated in these studies [7, 9, 50]. While it was difficult to explain variation in the relative risk between studies, it probably matters little to the conclusion that diabetes dramatically increases the incidence rate of PFA.

**Fig. 5:**
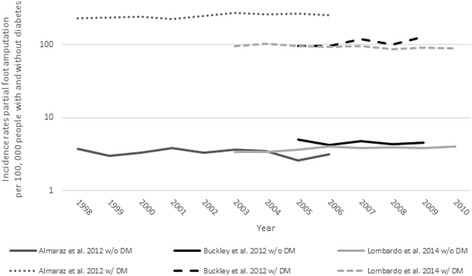
Incidence rate of partial foot amputation per 100,000 people with (w/) and without (w/o) diabetes

Three studies tested for changes in the incidence of PFA per 100,000 people with and without diabetes over time using either Poisson regression [9, 39] or Cuzick’s test; a non-parametric test for trends across three or more ordinal groups, presumed to be age categories [50]. For people with diabetes, two studies [9, 50] showed no statistically significant change in the incidence rate of PFA over time (Fig. 5). By contrast, Almaraz et al. [39] reported a 1.7% annual increase in the risk of PFA, which was statistically significant (RR 1.017, 95% CI 1.007–1.027, *p* = 0.001). We hypothesize that these different findings likely reflect that Almaraz et al. [39] excluded people younger than 30 years of age and therefore better captured the effect of time in an at-risk population, consistent with the increased RR of PFA as people with diabetes get older [9, 39]. For people without diabetes, the incidence rate of PFA was fairly consistent across studies and stable over time (Fig. 5). While two studies showed no statistically significant changes over time [39, 50], Lombardo et al. [9] reported a statistically significant increase in PFA over time (rate ratio 1.02, 95% CI 1.01 to 1.03, *p* < 0.001); in part, because the Poisson regression model was better able to control unexplained variance given that sex and age categories were included as independent variables and an interaction term was fitted to determine if the rate ratio changed over time.

### Incidence rate of PFA per 100,000 people with diabetes

The incidence rate of PFA per 100,000 people with diabetes were reported for 6 studies given standardization to a common denominator [43, 45, 46, 51–53].

**Fig. 6:**
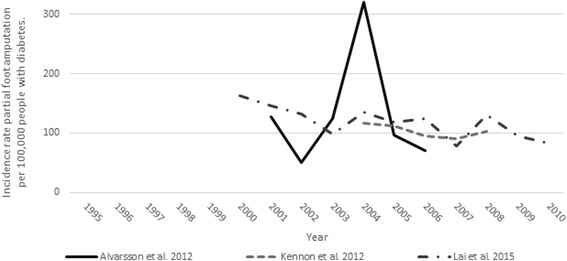
Incidence rate of partial foot amputation per 100,000 people with diabetes

Of these studies, five [45, 46, 51–53] included complete data that enabled a point estimate and confidence interval to be calculated (Table 7). The weighted mean annual incidence rate of PFA was 109.63 per 100,000 people with diabetes (95% CI 96.29 to 122.96), which was similar to that reported for the diabetes cohorts in studies that compared the incidence rate of PFA with and without diabetes [7, 9, 50]. The similarity of the mean annual incidence rate and relatively narrow 95% CI, masks the year-to-year variability observed in studies that reported data from a local healthcare service [45, 46, 52] where small numbers of amputations were performed each year. For example, Alvarsson et al. [52] reported dramatic swings in the incidence rate data (i.e., more than 50% increase or decrease in any year) that could be attributable to chance given there were an average of 6 amputations per annum over the time series (Fig. 6).

**Table 7:** Mean annual incidence rate of partial foot amputation per 100,000 people with diabetes

Study	Time period	Strata	Standardization			Annual incidence rate		Amputations per annum
			Crude	Age	Sex	Mean	SD	Mean
No Stratification								
Alvarsson et al. [52]	2001–2006		Yes	No	No	131.90	96.92	6
Canavan et al. [43]	1995–1999^a^		No	Yes	Yes	NR	NR	NR
Kennon et al. [51]	2004–2008		Yes	No	No	103.80	11.30	205
Lai et al. [53]	2000–2010		Yes	No	No	118.91	26.79	71
Rayman et al. [45]	1997–1999		Yes	No	No	123.33	27.15	11
Valabhji et al. [46]	2004–2008		Yes	No	No	146.64	85.64	7

^a^NR not reported. Data in article reported for 3 of 5 years, as such, it was impossible to calculate mean incidence rate for duration of the study

Three studies were designed to test whether the incidence rate of PFA per 100,000 people with diabetes changed over time; each used the chi-square for trend [51–53]. Given the relatively small number of amputations per annum in these studies, and the large fluctuations in the annual incidence rates as a result [51–53], it was not suprising there were no statisically significant changes in the incidence rate of PFA over time. While it would be easy to be critical of the choice of the chi-squared test for trend given that the incidence rates over time did not change linearly, we suggest that more sophisticated inferential analysis techniques would have been unlikely to yield a different result given the variability in the descriptive data reported (Fig. 6).

### Incidence rate of PFA versus TTA

Four studies reported incidence rate data for cohorts with dyscvascular PFA and TTA [13, 36, 48, 49]. None used inferential analysis to compare incidence rates between groups based on amputation level. Additionally, incidence rates were expressed in a variety of ways that did not facilitate synthesis. For example, of the two studies that reported incidence rates using the same denominator [13, 48], the levels of PFA included in these studies differed and one study was stratified by race [48]; thus tipifying the hetrogeneity between studies.

There was considerable variability in how the incidence rates of PFA and TTA compared between studies (Table 8). When people with toe amputation were included in the PFA cohort—either as a toe-only group or in combination with other levels of PFA—the incidence rates were similar to those observed in the TTA cohort [48, 49] (Table 8). Given that about two-thirds of all PFA are toe amputations, it is perhaps not suprising that when excluded from the PFA cohort, the incidence rates were approximately one-third that observed in the TTA cohort [13]. Given that few PFAs occur at the tarsometatarsal or transtarsal levels, their exclusion did not seem to affect how similar the incidence rates were between the PFA and TTA cohorts [49].

**Table 8:** Incidence rate of partial foot and transtibial amputation

Study	Time period	Strata	Standardization			Annual PFA incidence rate		Annual TTA incidence rate		Amputations per annum
			Crude	Age	Sex	Mean	SD	Mean	SD	Mean
Per 100,000 population										
Dillingham et al. [48]	1986–1997	Black race	No	Yes	Yes	19.00^a^	1.63	17.84	2.12	PFA 246; TTA 232
		Non-black race	No	Yes	Yes	12.78^a^	1.47	8.94	0.86	PFA 477; TTA 342
Feinglass et al. [13]	1987–2004		No	Yes	Yes	4.02^b^	0.93	10.57	1.12	PFA 319; TTA 822
Per 100,000 person-years general population										
Sandnes et al. [49]	1987–2000		No	Yes	Yes	7.93^c^	1.06	9.58	1.08	PFA 317; TTA 350
Per 1000 patient-years										
Davis et al. [36]	1993–2005		Yes	No	No	3.7	NR	1.1	NR	PFA 3; TTA 1

PFA Incidence rate includes data for toe amputation (3.4) and “foot” amputation (0.3); *NR* not reported. Unable to be calculated because there was no annual incidence rate data over time reported in the article

^a^PFA cohort includes only those with toe amputation

^b^PFA cohort includes “through foot” level only, excludes persons with only toe amputation

^c^PFA cohort includes people with toe/TMA amputation

Two studies described changes in the incidence rate over time for both a PFA and TTA cohort, using either Poisson [49] or linear regression (i.e., Wald test to determine whether the regression coefficient for time was statistically significant) [48]. Statistically significant increases in the incidence rates were observed in the TTA as well as the toe [48] or toe/transmetatarsal [49] amputation cohorts. While these studies were not designed to compare changes in the incidence rates over time between the PFA and TTA cohorts, one study [49] reported the annual incidence rate change and 95% CI for the toe/transmetatarasl (0.01%/year, 95% CI 0.01–0.02%) and transtibial (0.008%/year, 95% CI 0.003–0.0127%) amputation cohorts. Overlap of the 95% CI between the toe/transmetatasal and TTA cohorts indicates that changes over time were comparable [49]. It is important to note that both studies [48, 49] reported data for lengthy time series that ceased before the turn of the last centrury and, as such, there is some uncertainty about their representativeness in more contemporary healthcare settings.

### Prevalence

One study reported the prevalence of PFA [2], specifically amputation of the toe(s). In 2005, it was estimated that 302,000 Americans lived with amputation of the toe(s). It is important to recognize that the estimated prevalence was based on incidence rate data collected from hospital discharges from 1988 to 1999. Given the proportion of hospital discharges due to reamputation, it was prudent that the estimated prevalence was reduced by 26% to better reflect the number of people affected. Despite the assumption that the incidence rate would remain constant over time, and that historical trends in population and mortality would hold true into the future, we should have some confidence in the prevalence estimate for the year 2005 given the proximity to the data collection period. Given that prevalence data were not estimated beyond 2005 for people with PFA, concerns with accuracy of the longer-term prevalence predictions are not relevant to this review.

## Discussion

The purpose of the review was to develop an informed understanding of the incidence rate and prevalence of dysvascular PFA, how these compared to TTA, and how they have changed over time. To the best of our knowledge, this is the first systematic review that has endeavored to synthesize the incidence rate and prevalence data of any type of lower limb amputation. While systematic reviews of epidemiological data are common in many areas of healthcare, they have typically only been performed in well-developed bodies of literature where numerous studies report incidence rates using the same denominator, thereby facilitating synthesis using meta-analysis. We suggest that systematic reviews of epidemiological data in lower limb amputation have not previously been undertaken given the wide variety of methodological and reporting approaches that make the literature too heterogeneous to synthesize using statistical approaches. However, this does not negate the need for a clear understanding about the incidence rate or prevalence and how these have changed over time.

As highlighted in this review, the many different methodological and reporting approaches added considerably to the challenge of synthesizing data. We struggled to bring together data from such disparate studies until we recognized that we could reduce much of the apparent variation between studies by standardizing the incidence rates to common denominators; something only evident to us after careful critique of the way the denominators were operationally defined. By standardizing the incidence rates to common denominators where appropriate, we were able to reduce the apparent variation between studies that, in turn, made it possible to glean new insights into these studies leading to a more informed understanding.

In contrast to our initial impression of the literature, we were surprised by how homogenous the incidence rate were once studies were appropriately grouped by the same denominator and strata. We do not imply that there is absolute agreement between studies, just less variation than might be expected based on a primafacie evaluation of the epidemiological literature.

There were examples where the incidence rates of PFA were very homogenous or where variation between studies did not reduce confidence in the conclusions. For example, studies that reported the incidence rate of PFA per 100,000 general population had a very narrow confidence interval (4.0 per 100,000 general population, 95% CI, 3.82 to 4.17). While there was less precision in the incidence rate of PFA for people with diabetes (94.24 per 100,000 people with diabetes; 95% CI 55.50 to 133.00) compared to those without (3.80 per 100,000 people without diabetes; 95% CI 1.43 to 6.16), it probably matters little to the conclusion that risk of PFA is significantly greater (about 25 times greater) for people with diabetes [7, 9, 50].

In terms of diabetes type, the higher incidence rates typically associated with type 2 diabetes [42, 47] may be confounded by inclusion of people with first and recurrent amputation in the same cohort. When stratified by both diabetes type and initial/recurrent amputation, incidence rates of initial PFA were similar in cohorts with types 1 and 2 diabetes [38]. In comparison, the incidence rate of recurrent PFA was 23 times higher in people with type 1 diabetes and 100 times higher in people with type 2 diabetes [38]; highlighting the very high risk of recurrent PFA, particularly in people with type 2 diabetes.

The inclusion or exclusion of people with amputation of the toe(s) had a profound effect on the incidence rate of the PFA cohort, which may not be suprising given the majority of PFA affects one or more toes. By comparison, the inclusion or exclusion of people with more proximal level of PFA (e.g., tarsometatarsal or transtarsal amputation) did not seem to have a dramatic effect on the incidence rates.

When people with toe amputation were included in the PFA cohort, the incidence rates were comparable to TTA [48, 49]. Given the time period during which these data were collected, there is uncertainty about the generalizability of these results to more contemporary healthcare settings.

There is little certainty in whether the incidence rates of PFA have remained stable over time or changed. To some extent, the uncertainty reflects the small number of like studies when considered with respect to the different denominators and strata. A number of common method design issues further complicated our understanding. Time series were often too short for small changes in the annual incidence rate of PFA—typically less than 1–2% per annum—to become sufficiently large to be statistically significant. More dramatic reductions in the annual incidence rate of PFA reported in the literature should be interpreted with caution given they were able to be explained by common method design issues such as the difficulties in accurately estimating diabetes prevalence or inappropriate inferential analyses. Studies with small subject numbers were suseptible to chance variation in the number of cases in any given year and, as such, the year-to-year variability tended to dwarf any small, cumulative, change in the annual incidence rate. While most studies used inferential analysis techniques designed to test for trends in epidemiological data, there was often little consideration about the suitability of the statistical tests given the descriptive data presented. For example, the chi-square test for trend can exaggerate the significance of the change over time where the annual incidence rate were not linear. Given the uncertainty introduced by these method design issues, it is unclear whether the incidence rate of PFA has changed over time or how this compares to changes over time in those with TTA.

As the first systematic review to synthesize epidemiological data describing the incidence rate and prevalence of PFA, we have been able to benchmark what could be considered typical incidence rates for each of the common denominators and strata. These data may be particularly valuable where sufficient studies made it possible to calculate a point estimate and 95% CI.

### Future research

Given the insights gleaned from this review, we suggest that there is little opportunity to extend our understanding based on epidemiological studies of isolated hospitals, short-time series, and small numbers of amputations per annum. Large-scale epidemiological studies over lengthy time series are required. In all likelihood, these studies will require linked datasets of state or national amputation surgeries that include the thousands of people per annum needed to stratify by important risk factors. Only in this way, can we corroborate insights about the influence of diabetes type, initial/recurrent amputation or race, and thereby clarify our understanding.

In the same vein, studies that test for trends over time should ensure that the assumptions of the inferential analysis match the descriptive data and consider more sophisticated approaches, such as joinpoint regression, that may better test changes in trends within the time series. More contemporary prevalence estimate are also desperately needed, as are studies that stratify by race.

### Limitations

Given the myriad of ways the incidence rates were reported in the literature, we made a number of pragmatic decisions to be able to synthesize the results across like studies. We collapsed crude or age- and sex-standardized incidence rate data given that in the two studies [9, 37] that reported both these data, the incidence rates were highly correlated (*r* > 0.98), suggesting that, for these two studies, and probably others, combining crude and standardized incidence rates would be unlikely to change our observations.

We standardized like denominators (e.g., incidence rate per 10,000 or per 100,000 general population) to reduce apparent variation between studies. Where we have done so, we have made it clear in the results narrative given the absolute incidence rates reported in the review will differ from those reported in the original research. We argue that this approach reduced apparent variation that made it possible to see the similarity in incidence rates across studies and identify true, not apparent, sources of variation.

We felt the only way to present these incidence rates were by subgroup within each denominator and thereby preserve information inherent in the different strata. Given the number of subgroup analyses, and the detailed narratives contextualizing the risk of bias, we did not feel it was necessary to include the risk of bias tables within the body of the manuscript; particularly given the McMaster Critical Review forms included a written appraisal of each article to supplement the check list items. We have reported the complete risk of bias assessment for each article as part of the appendix for readers wanting this level of detail (Additional file 1).

## Conclusion

Incidence rate data were quite homogenous when studies were appropriately grouped by the same denominator and strata. Where there was variation between studies, it often did not alter the conclusions. For example, variation in the incidence rate of PFA in people with diabetes compared to without diabetes, probably matters little given the conclusion that diabetes increased the relative risk about 25 times. The higher incidence rates typically associated with type 2 diabetes may be confounded by inclusion of people with first and recurrent amputation in the same cohort. There is little certainty in whether the incidence rates of PFA have remained stable over time or changed. There were common biases that reflected small samples with large year-to-year variability that masked small cumulative changes in the incidence rates reported. Similarly, inferential analysis techniques were often not appropriate given violations of their assumptions. Further research using state or national datasets that includes large numbers of amputations each year, and lengthy time series, are needed to determine whether the small annual changes in incidence rates have a cumulative and statistically significant effect over time.

## Additional file

Additional file 1: Data extraction and risk of bias assessment for included studies. (XLSX 69 kb)

## Abbreviations

ISO: International Standards Organization
MeSH: National Library of Medicine, Medical Subject Headings
PFA: Partial foot amputation
PRISMA: Preferred Reporting Items for Systematic Reviews and Meta-Analyses
TTA: Transtibial amputation
